# Correlation Between Systemic Inflammation Response Index and Systemic Immune-Inflammation Index and Disease Activity of Lupus Nephritis in Children

**DOI:** 10.3390/children13040530

**Published:** 2026-04-11

**Authors:** Desi Mutiarati, Gartika Sapartini, Sri Endah Rahayuningsih, Dany Hilmanto, Eddy Fadlyana, Dedi Rachmadi

**Affiliations:** Department of Child Health, Faculty of Medicine, Universitas Padjadjaran, Hasan Sadikin General Hospital, Bandung 40161, Indonesia; desi21005@mail.unpad.ac.id (D.M.); sri.endah@unpad.ac.id (S.E.R.); dany.hilmanto@unpad.ac.id (D.H.); eddy.fadlyana@unpad.ac.id (E.F.); dedi.rachmadi@unpad.ac.id (D.R.)

**Keywords:** lupus nephritis, disease activity, systemic inflammation response index, systemic immune-inflammation index, Mex-SLEDAI, children

## Abstract

**Objectives**: Lupus nephritis (LN) is a severe manifestation of pediatric systemic lupus erythematosus (SLE) requiring accurate disease activity assessment. This study evaluated the association of the Systemic Inflammation Response Index (SIRI) and the Systemic Immune-Inflammation Index (SII) with disease activity in children with LN. **Methods**: In this cross-sectional study, 52 children with LN aged 1 month to 18 years treated at Dr. Hasan Sadikin General Hospital, Indonesia, were included. SIRI and SII were calculated from complete blood counts, and disease activity was assessed using the Mexican Modification of the SLE Disease Activity Index (Mex-SLEDAI). Correlations were analyzed using Spearman’s test. **Results**: Median SIRI and SII values were 1511 and 789,544, respectively, with a median Mex-SLEDAI score of 7. SIRI showed a moderate positive correlation with disease activity (r = 0.443; *p* < 0.001), and SII also showed a significant positive correlation (r = 0.390; *p* = 0.004). Both indices increased with higher LN activity. **Conclusions**: SIRI and SII were significantly associated with disease activity in pediatric LN, with SIRI yielding a numerically higher correlation coefficient, though this difference was not formally compared. These indices may serve as simple, non-invasive biomarkers for assessing inflammatory activity in children with lupus nephritis.

## 1. Introduction

Systemic lupus erythematosus (SLE) is a rare, yet severe chronic autoimmune disease characterized by persistent inflammation and multisystem involvement, frequently resulting in irreversible organ damage [1]. The pathogenesis of SLE is driven by profound immune dysregulation, including loss of immune tolerance, autoantibody production, immune complex deposition, and sustained activation of inflammatory pathways, ultimately leading to dysfunction across multiple organ systems [2,3]. One of the most common and serious complications of childhood-onset SLE is renal involvement, also referred to as lupus nephritis (LN) [4,5].

LN is a form of immune complex-mediated glomerulonephritis resulting from the deposition of circulating immune complexes within the renal glomeruli as part of the autoimmune process underlying SLE [6]. LN occurs more frequently in pediatric-onset SLE than in adult-onset disease, with a reported prevalence of 50–82% in children compared with 20–40% in adults [7]. Besides being more prevalent, LN in children is usually linked to a more aggressive course of the illness and a significantly greater risk of long-term renal impairment [7]. Notably, with reported frequencies of up to 88% during the first year and 93% within the second year following SLE diagnosis, LN frequently marks the earliest clinical manifestation of SLE in children [8]. Clinical and laboratory results, such as hematuria, proteinuria > 0.5 g/24 h or a urine protein-to-creatinine ratio > 0.5 mg/mg, hypertension, and decreased glomerular filtration rate, reveal an increased likelihood of renal involvement [9]. Renal biopsy is advised in these situations to determine histological categorization and to guide prognostic stratification [6].

Despite breakthroughs in immunosuppressive medication and supportive care that have improved overall survival, LN remains a key contributor to morbidity and death in pediatric SLE [10]. About 15% of people with SLE, especially those with severe histologic subtypes, develop end-stage renal disease (ESRD), and about 70% of children with SLE develop LN [7,11]. These findings underline the essential necessity of early detection of renal involvement and precise evaluation of disease activity, particularly within the first three years following SLE diagnosis [12].

SLE progression is indicative of a dynamic inflammatory state that may be reversed with prompt and suitable immunomodulatory treatment [13]. Since prolonged activity or illness flare-ups are significantly linked to unfavorable clinical outcomes, such as irreversible organ damage, higher mortality, and worse quality of life, accurate evaluation of disease activity is crucial [14]. The Systemic Lupus Erythematosus Disease Activity Index (SLEDAI) and its simplified formulation, the Mexican Modification of the Systemic Lupus Erythematosus Disease Activity Index (Mex-SLEDAI), are among the most used tools for evaluating SLE disease activity [15]. Mex-SLEDAI is particularly useful in situations with low resources since it emphasizes clinical signs and does not include complement and anti-double-stranded DNA tests [16]. Significantly, the Mex-SLEDAI scoring system gives renal involvement a high weight, indicating its crucial significance in assessing the severity of the illness [17]. A Mex-SLEDAI score ≥ 5 indicates active disease, with reported sensitivity and specificity of 87.5% and 100%, respectively [18].

LN is frequently assessed using standard laboratory markers, such as proteinuria, serum creatinine, blood urea nitrogen, anti-dsDNA antibodies, and complement levels [19]. However, these markers exhibit limited sensitivity and specificity and often fail to distinguish between active inflammatory renal injury and chronic irreversible damage [20]. In pediatric LN, this is a particularly important limitation, since early identification of ongoing inflammatory activity may allow clinicians to adjust treatment before irreversible organ damage accumulates. It should also be considered that complement and anti-dsDNA testing are not always available or affordable in resource-limited settings, which represent the clinical context for many pediatric LN patients. Moreover, emerging molecular biomarkers such as cytokines and chemokines, while mechanistically informative, are influenced by genetic background, environmental exposure, and immune status, limiting their generalizability and routine clinical use [21]. Consequently, there remains a need for simple, accessible, and non-invasive biomarkers that can dynamically reflect disease-related inflammatory activity in pediatric LN [11].

Hematological parameters derived from routine complete blood counts have gained increasing attention as surrogate markers of systemic inflammation and disease activity across a range of inflammatory and autoimmune disorders, including SLE [22]. The Systemic Inflammation Response Index (SIRI) and the Systemic Immune-Inflammation Index (SII) integrate multiple circulating immune cell populations, thereby capturing the interplay between innate immune activation and adaptive immune regulation [23]. These indices have demonstrated diagnostic and prognostic value in various inflammatory and autoimmune conditions [21]. From a pathophysiological perspective, neutrophils contribute to SLE progression through the formation of neutrophil extracellular traps (NETs), which amplify inflammation and tissue injury, while lymphocytes, monocytes, and platelets participate in autoantibody production, cytokine signaling, endothelial dysfunction, and vascular damage that accompany active disease [24].

Although several studies in adult populations have highlighted the potential utility of SIRI and SII as biomarkers of LN activity, evidence in pediatric cohorts remains limited and inconsistent. Validation of these indices against standardized disease activity instruments such as the Mex-SLEDAI has not been adequately explored in children. Therefore, this study aims to investigate the correlation between SIRI, SII, and disease activity in children with LN as an initial step toward validating simple, non-invasive, and clinically applicable inflammatory biomarkers for monitoring disease activity and preventing long-term renal complications in pediatric LN.

## 2. Materials and Methods

### 2.1. Study Design and Participants

This observational analytical study employed a cross-sectional design to examine the association between systemic inflammatory indices and disease activity in pediatric LN. The study was conducted at Hasan Sadikin General Hospital, Bandung.

Children aged > 1 year and <18 years with a confirmed diagnosis of LN according to the 2012 Systemic Lupus International Collaborating Clinics (SLICC) classification criteria or the 2019 European League Against Rheumatism/American College of Rheumatology (EULAR/ACR) criteria were eligible for inclusion [25]. Exclusion criteria comprised a history of autoimmune diseases other than SLE, malignancy, severe hepatic disease, severe cardiovascular or cerebrovascular disease, and other conditions known to cause renal impairment or proteinuria, including diabetic nephropathy, nephrotic syndrome, multiple myeloma, or amyloidosis.

### 2.2. Sample Size and Enrolment

The sample size was calculated to detect a correlation between SIRI, SII, and LN disease activity measured using the Mex-SLEDAI. Assuming a two-sided significance level (α) of 0.05, statistical power of 80%, and a moderate correlation coefficient (r = 0.4), a minimum sample size of 47 participants was required. In the absence of prior pediatric-specific data, this effect size was chosen conservatively. To account for potential incomplete or missing data, the sample size was increased by 10%, resulting in a final target of 52 participants.

Participants were enrolled using consecutive sampling, whereby all eligible patients presenting during the study period were recruited sequentially until the target sample size was reached.

### 2.3. Variables and Measurements

The primary exposure variables were SIRI and SII. SIRI was calculated as neutrophil count × monocyte count/lymphocyte count, and SII as neutrophil count × platelet count/lymphocyte count. All hematological parameters were obtained from routine complete blood count analyses performed as part of standard clinical care. The measurements of hematological parameters were done by using Sysmex XN-1000 machine.

The primary outcome was LN disease activity, assessed using the Mex-SLEDAI score (range 0–32). Disease activity was categorized as mild (2–5), moderate (6–9), or severe (≥10). Nutritional status was evaluated as a potential confounding variable. In children younger than 5 years, nutritional status was assessed using weight-for-height or weight-for-length indices according to the 2006 World Health Organization (WHO) growth standards. In children aged 5–18 years, nutritional status was assessed using body mass index-for-age based on the 2007 WHO reference standards.

### 2.4. Data Collection

Eligible participants were identified through systematic review of medical records. Data extracted included demographic characteristics, clinical features, and laboratory findings. Mex-SLEDAI scores were calculated using contemporaneous clinical and laboratory data. SIRI and SII values were derived from complete blood count results obtained at the same clinical encounter.

### 2.5. Statistical Analysis

Statistical analyses were performed using Stata version 17. All statistical tests were two-sided, and a *p* value ≤ 0.05 was considered statistically significant. Continuous variables are presented as mean ± standard deviation for normally distributed data or as median with interquartile range for non-normally distributed data, as assessed using the Shapiro–Wilk test. Categorical variables are reported as frequencies and percentages. Associations between SIRI, SII, and LN disease activity were evaluated using correlation analyses. Pearson’s correlation coefficient was applied for normally distributed variables, whereas Spearman’s rank correlation coefficient was used for non-normally distributed variables. Correlation strength was interpreted according to standard thresholds

## 3. Results

### 3.1. Participant Characteristics

A total of 52 pediatric patients with LN who met the inclusion and exclusion criteria were included in the analysis. Baseline characteristics are summarized in Table 1. The mean age of participants was 14.1 ± 2.9 years, and most were female (86.5%). The majority of patients had normal nutritional status (80.8%). Moderate malnutrition was observed in 7.7% of participants and severe malnutrition in 5.8%, while 3.8% were overweight and 1.9% were obese.

The median of SIRI was 1511 (range, 206–42,925), and the median of SII was 789,544 (range, 4000–7,085,463). The median Mex-SLEDAI score was 7 (interquartile range, 2–13). Based on disease activity categories, 55.8% of patients had moderate disease activity, 28.8% had mild activity, and 15.4% had severe activity.

### 3.2. Correlation Between Inflammatory Indices and Disease Activity

Correlation analyses demonstrated significant positive associations between both inflammatory indices and LN disease activity. SIRI showed a moderate positive correlation with Mex-SLEDAI scores (r = 0.443, *p* < 0.001). SII was also positively correlated with Mex-SLEDAI scores (r = 0.390, *p* = 0.004). While SIRI had a numerically higher correlation coefficient, the difference between the two was not formally tested.

**Figure 1:**
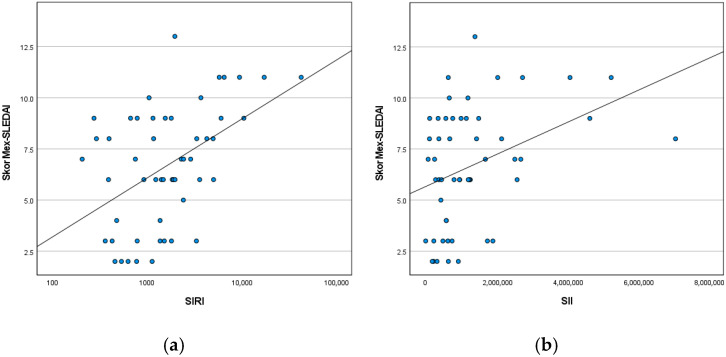
Scatterplot of the relationship between (**a**) SIRI and (**b**) SII and Mex-SLEDAI scores in children with LN.

Scatterplot analyses demonstrated a positive relationship between SIRI and Mex-SLEDAI scores, with higher disease activity observed at higher SIRI values (Figure 1a). A similar positive trend was observed between SII and Mex-SLEDAI scores (Figure 1b); however, the distribution of data points was more dispersed, consistent with the weaker correlation observed for SII.

### 3.3. Inflammatory Indices Across Disease Activity Categories

SIRI and SII values differed significantly across disease activity categories (Table 2). Median SIRI values increased progressively with disease severity: 789 (362–3342) in mild activity, 1564 (206–10,615) in moderate activity, and 6201 (1054–42,925) in severe activity (Kruskal–Wallis test, *p* = 0.001).

A similar pattern was observed for SII. Median SII values were 585,870 (4000–1,908,444) in mild activity, 968,568 (73,489–7,085,463) in moderate activity, and 1,722,742 (644,318–5,261,176) in severe activity, with a statistically significant difference across groups (*p* = 0.010).

## 4. Discussion

LN remains one of the most severe manifestations of SLE, contributing substantially to morbidity and mortality, particularly in pediatric populations [26]. Proliferative LN in children progresses to end-stage renal disease within five years in approximately 9–15% of cases [27], and mortality among juvenile-onset SLE patients is nearly twice that of adult-onset disease, as demonstrated in the Lupus in Minorities: Nature vs. Nurture (LUMINA) cohort [28]. In this context, identifying simple, accessible biomarkers capable of reflecting disease activity is of critical clinical importance. The present study demonstrates that systemic inflammatory indices, specifically the SIRI and the SII, are significantly associated with LN disease activity in children.

The demographic characteristics of the study population align with established epidemiological patterns of pediatric LN. The marked female predominance (86.5%) and adolescent age distribution are consistent with previous reports indicating that LN is more common in females and often presents during adolescence [29]. A previous study similarly reported that approximately 80% of pediatric LN patients were female, with a median age at diagnosis of around 11 years [30]. Female predominance in LN has been attributed to a combination of hormonal, genetic, and environmental factors [31]. Puberty-related increases in estrogen exert immunomodulatory effects, including enhanced B-cell activation, increased autoantibody production, and reduced immune tolerance [32]. In addition, disrupted X-chromosome epigenetic regulation has been implicated in lupus susceptibility, further contributing to sex-based differences in disease risk [33].

Most participants in this study had normal nutritional status, consistent with previous findings, which observed no strong association between simple anthropometric categories and disease activity in pediatric SLE [34]. The predominance of moderate disease activity based on Mex-SLEDAI scores is also consistent with prior regional studies, including data indicating that most pediatric LN patients present with mild to moderate activity [35]. The Mex-SLEDAI places substantial weight on renal manifestations [36]. Thus, renal involvement itself directly contributes to higher disease activity scores. Moreover, the inclusion of patients receiving ongoing therapy likely contributed to the relatively lower proportion of severe disease activity observed in this cohort.

A key finding of this study is the moderate positive correlation between SIRI and LN disease activity. SIRI reflects the balance between innate immune and adaptive immune regulation, reflected by lymphocyte counts. In active LN, dysregulated innate immune responses play a central role in mediating renal injury [33,37]. Excessive activation of neutrophils and monocytes promotes immune complex deposition, complement activation, and amplification of local inflammatory cascades within the glomerulus [38].

Neutrophils contribute to LN pathogenesis through the formation of neutrophil extracellular traps (NETs), which contain DNA, histones, and cytotoxic proteins [39]. NETs promote immune complex formation, complement activation, and glomerular inflammation [40]. Concurrently, renal monocytes and macrophages exacerbate tissue injury through the production of pro-inflammatory cytokines such as interleukin-6 and tumor necrosis factor-α [41]. These innate immune processes are accompanied by relative lymphopenia, a hallmark of active SLE, driven by increased lymphocyte apoptosis and chronic immune consumption [42]. Together, these mechanisms explain the progressive increase in SIRI observed with rising LN disease activity [43,44].

In addition to SIRI, this study demonstrates a significant association between SII and LN disease activity. SII integrates neutrophil, platelet, and lymphocyte counts, thereby reflecting the interplay between systemic inflammation, immune dysregulation, and thromboinflammatory processes [21,45]. Platelets are increasingly recognized as active participants in immune responses, contributing to leukocyte activation, cytokine release, endothelial dysfunction, and microvascular injury [46]. In LN, heightened platelet activation may exacerbate renal inflammation and impair microcirculation, contributing to elevated SII values during active disease [21].

Lymphopenia, a common hematological feature of active SLE, further amplifies SII values and correlates with higher Mex-SLEDAI scores [17]. Reduced peripheral lymphocyte counts are thought to result from increased apoptosis induced by inflammatory cytokines, as well as redistribution of lymphocytes to inflamed tissues [47]. The combined effect of neutrophilia, thrombocytosis, and lymphopenia provides a plausible biological basis for the observed association between SII and LN disease activity [48].

The findings of this study are consistent with reports from adult populations. A study demonstrated higher SII values in LN patients compared with SLE patients without proteinuria, with significant correlations with SLEDAI scores [49]. SII was identified as an independent predictor of LN and reported associations with SLEDAI-2K scores [21], while SII was shown to be correlated with clinical markers such as proteinuria, serum creatinine, and renal pathological activity indices [50]. However, some studies suggest that SII may be more reflective of current inflammatory burden rather than a reliable predictor of short-term remission, highlighting the need for longitudinal evaluation [23].

It is important to consider that several factors other than disease activity itself may have influenced the SIRI and SII values observed in this study. Most patients with LN receive corticosteroids and immunosuppressive agents as part of their treatment regimen, and these medications are known to affect circulating leukocyte counts. Corticosteroids, for instance, typically cause neutrophilia and lymphopenia through demargination and redistribution, which would tend to increase both SIRI and SII independently of the underlying inflammatory state. Immunosuppressive drugs such as mycophenolate mofetil and cyclophosphamide can suppress bone marrow function, leading to leukopenia or lymphopenia that may also alter the index values. In addition, concurrent infections, which are common in immunosuppressed patients, can cause reactive changes in neutrophil and monocyte counts that would further confound the association between these indices and disease activity. We did not stratify the analysis by medication type or dosage, nor did we exclude patients with documented infections at the time of blood sampling, which means that the observed correlations may partly reflect treatment effects or infectious comorbidity rather than LN activity alone. This is an important consideration when interpreting the results, and future studies should attempt to account for these confounders either through stratified analysis or multivariable regression models.

Another aspect that deserves consideration is the absence of a control group in this study. We did not include healthy children or SLE patients without nephritis for comparison, which means that we cannot determine whether the elevated SIRI and SII values are specific to LN or simply reflect the systemic inflammatory burden of SLE in general. It is possible that SLE patients without renal involvement may also have elevated values of these indices, and without such a comparison group, the specificity of SIRI and SII as markers of nephritis activity, rather than SLE activity overall, remains uncertain. Similarly, we did not have data on remission and flare status, which would have been useful to assess whether these indices change meaningfully during different disease phases. These comparisons would be valuable to explore in future studies to better characterize the clinical context in which SIRI and SII may be most informative.

Overall, the present study indicates that both SIRI and SII are significantly associated with LN disease activity in children. SIRI had a numerically higher correlation coefficient compared to SII, although the two coefficients were not formally compared and therefore this difference should not be overinterpreted. The findings are consistent with the role of innate immune activation, particularly neutrophils and monocytes, in the pathogenesis of pediatric LN. Given their simplicity, low cost, and availability from routine blood tests, SIRI and SII may serve as useful adjunctive biomarkers for assessing disease activity in pediatric LN.

Several limitations should be acknowledged. The cross-sectional design precludes assessment of temporal relationships and causality between inflammatory indices and disease activity. In this study, the associations were evaluated using univariate correlation analysis, and we did not perform multivariable adjustment for potential confounders, including the type and intensity of immunosuppressive therapy, the presence of concurrent infections, or SLE-related hematologic abnormalities such as autoimmune cytopenias. Given the sample size of 52 patients, even a simple regression model adjusting for age, sex, and disease duration would have helped to clarify whether the observed correlations are independent, and we recognize this as a shortcoming of the current analysis. We also note that SIRI had a numerically higher correlation coefficient compared to SII, but this difference was not compared using a formal statistical test such as Steiger’s Z-test; accordingly, claims about whether one index performs better than the other should be made with caution. As discussed above, the lack of a control group—whether healthy children, SLE patients without nephritis, or patients in remission—limits our ability to determine whether elevated SIRI and SII values are specific to LN or reflect SLE-related inflammation more broadly. Another limitation is the considerable overlap in SIRI and SII values between disease activity categories, which can be seen from the wide ranges reported in each group. We did not perform receiver operating characteristic analysis or define cutoff values, and therefore, it remains unclear how well these indices can discriminate between individual patients with different levels of disease activity. The sample size was also relatively small, and the study was conducted at a single center, which may affect the generalizability of the results. Longitudinal multicenter studies with larger samples, appropriate control groups, and multivariable analysis are needed to confirm whether SIRI and SII have robust and clinically meaningful utility in pediatric LN.

## 5. Conclusions

This study demonstrates that SIRI and SII are significantly associated with LN disease activity in children, as assessed by the Mex-SLEDAI score. Both indices increase in parallel with disease severity. SIRI had a numerically higher correlation coefficient than SII, although this difference was not formally compared. These findings support the role of innate immune activation, particularly involving neutrophils and monocytes, in the pathophysiology of pediatric LN. As SIRI and SII are derived from routine complete blood counts, they represent simple, non-invasive, and readily accessible biomarkers that may complement existing tools for assessing disease activity, particularly in resource-limited clinical settings. However, further validation through longitudinal and multivariable studies is needed to confirm the clinical utility of these indices and to establish meaningful cutoff thresholds.

## Figures and Tables

**Table 1:** Characteristic of the Subjects.

Characteristics	Value
Age (year), mean ± SD	14.1 ± 2.9
Sex, n (%)	
Male	7 (13.5)
Female	45 (86.5)
Nutritional status, n (%)	
Severe malnutrition	3 (5.8)
Moderate malnutrition	4 (7.7)
Normal	42 (80.8)
Overweight	2 (3.8)
Obese	1 (1.9)
SIRI, Median (Min–Max)	1511 (206–42,925)
SII, Median (Min–Max)	789,544 (4000–7,085,463)
Mex-SLEDAI score, Median (Min–Max)	7 (2–13)
Disease activity, n (%)	
Low	15 (28.8)
Moderate	29 (55.8)
High	8 (15.4)

Mex-SLEDAI = Mexican Modification of the Systemic Lupus Erythematosus Disease Activity Index, SII = systemic immune–inflammation index, SIRI = systemic inflammatory response index.

**Table 2:** Differences between SIRI and SII by disease activity category.

	Disease Activity	n	Median (Min–Max)	*p* Value
SIRI	Low	15	789 (362–3342)	0.001 *
	Moderate	29	1564 (206–10,615)	
	High	8	6201 (1054–42,925)	
SII	Low	15	585,870 (4000–1,908,444)	0.010 *
	Moderate	29	968,568 (73,489–7,085,463)	
	High	8	1,722,742 (644,318–5,261,176)	

* *p* < 0.05 on Kruskal–Wallis test. SII = systemic immune–inflammation index, SIRI = systemic inflammatory response index.

## Data Availability

The original contributions presented in this study are included in the article. Further inquiries can be directed to the corresponding author.

## Abbreviations

The following abbreviations are used in this manuscript:

ACR	American College of Rheumatology
EULAR	European League Against Rheumatism
ESRD	End-stage renal disease
LN	Lupus nephritis
LUMINA	Lupus in Minorities: Nature vs. Nurture
Mex-SLEDAI	Mexican Modification of the Systemic Lupus Erythematosus Disease Activity Index
NETs	Neutrophil extracellular traps
SII	Systemic Immune-Inflammation Index
SIRI	Systemic Inflammation Response Index
SLE	Systemic lupus erythematosus
SLEDAI	Systemic Lupus Erythematosus Disease Activity Index
SLICC	Systemic Lupus International Collaborating Clinics
WHO	World Health Organization
